# 99mTc-MIBI Lung Scintigraphy in the Assessment of Pulmonary Involvement in Interstitial Lung Disease and Its Comparison With Pulmonary Function Tests and High-Resolution Computed Tomography: A Preliminary Study

**DOI:** 10.1097/MD.0000000000002082

**Published:** 2015-10-30

**Authors:** Mehrzad Bahtouee, Jamshid Saberifard, Hamid Javadi, Iraj Nabipour, Alireza Raeisi, Majid Assadi, Mohammad Eftekhari

**Affiliations:** From the Department of Internal Medicine (Division of Pulmonary), Bushehr Medical Center Hospital (MB); Department of Radiology, Bushehr Medical Center Hospital, Bushehr University of Medical Sciences, Bushehr (JS); Golestan Research Center of Gastroenterology and Hepatology (GRCGH), Golestan University of Medical Sciences (GUOMS), Gorgan (HJ); The Persian Gulf Tropical Medicine Research Center, (IN, AR); The Persian Gulf Nuclear Medicine Research Center, Bushehr University of Medical Sciences, Bushehr (MA); and Research Center for Nuclear Medicine, Tehran University of Medical Sciences, Tehran, Iran (ME).

## Abstract

The differentiation of active inflammatory processes from an inactive form of the disease is of great value in the management of interstitial lung disease (ILD). The aim of this investigation was to assess the efficacy of 99mTc-methoxy-isobutyl-isonitrile (99mTc-MIBI) scans in distinguishing the severity of the disease compared to radiological and clinical parameters.

In total, 19 known cases of ILD were included in this study and were followed up for 1 year. Five patients without lung disease were considered as the control group. The patients underwent pulmonary function tests (PFTs) and high-resolution computed tomography scans, followed by 99mTc-MIBI scanning. The 99mTc-MIBI scans were analyzed either qualitatively (subjectively) or semiquantitatively.

All 19 ILD patients demonstrated a strong increase in 99mTc-MIBI uptake in the lungs compared to the control group. The 99mTc-MIBI scan scores were higher in the patient group in both the early phase (0.24[0.19–0.31] vs 0.11[0.10–0.15], *P* < 0.05) and the delayed phase (0.15[0.09–0.27] vs 0.04[0.01–0.09], *P* < 0.05) compared with the control group. A positive correlation was detected between the 99mTc-MIBI scan and the high-resolution computed tomography (HRCT) scores (Spearman's correlation coefficient = 0.65, *P* < 0.02) in the early phase but not in the delayed phase in patients (*P* > 0.14). The 99mTc-MIBI scan scores were not significantly correlated with the PFT findings (*P* > 0.05). In total, 5 patients died and 14 patients were still alive over the 1-year follow-up period. There was also a significant difference between the uptake intensity of 99mTc-MIBI and the outcome in the early phase (dead: 0.32[0.29–0.43] vs alive: 0.21[0.18–0.24], *P* < 0.05) and delayed phase (dead: 0.27[0.22–0.28] vs alive: 0.10[0.07–0.19], *P* < 0.05).

The washout rate was ∼40 min starting from 20 min up to 60 min and this rate was significantly different in our 2 study groups (ILD: 46.61[15.61–50.39] vs NL: 70.91[27.09–116.36], *P* = 0.04).

The present study demonstrated that 99mTc-MIBI lung scans might distinguish the severity of pulmonary involvement in early views, which were well correlated with HRCT findings. These results also revealed that 99mTc-MIBI lung scans might be used as a complement to other diagnostic and clinical examinations in terms of functional information in ILD; however, further investigations are strongly required.

## INTRODUCTION

Interstitial lung disease (ILD) and diffuse parenchymal lung disease are characterized by inflammation and fibrosis in the alveoli, distal airways, and septal interstitium of the lungs.^1–3^

Timely and precise identification of ILD can be challenging, but it is critical to the patient's management.^4,5^ Generally, the clinical impression is based on the integration of clinical, radiological, and histopathological features.^6^

Interstitial lung disease identification is ultimately launched by a lung biopsy, which is an invasive method, and is problematic to repeat during follow-up. Moreover, histological findings are not absolutely in accordance with the clinical severity of the disease.^7^ In most clinical scenarios no single investigation modality is perfect. Ideally a combination of modalities may be required to ascertain the best judgment.

Currently, high-resolution computed tomography (HRCT) and pulmonary function tests (PFTs) are the most commonly used noninvasive diagnostic tests for the assessment of ILD.^8^ Although the findings from the HRCT are related to the histological findings, its clinical effectiveness is hampered by the qualitative interpretation of the results.^9^ Furthermore, PFTs, which are well correlated to a biopsy and HRCT findings, are the most simple modality for follow-up studies.^10^ Nonetheless, their clinical value is debatable, especially when there are contradictory findings from the PFTs and HRCT.^11^ Hence, the ideal approach to judge pulmonary involvement in patients with ILD remains uncertain.

In nuclear medicine fields, 67Ga-citrate, 111In-octreotide, and 99mTc-diethylene triamine pentaacetic acid (99mTc-DTPA) aerosol for scintigraphic recognition of ILD have been proposed.^4,12–14^ However, an optimal modality with a high concordance with HRCT and PFT findings is still missing.

One lipophilic imaging agent that is widely applied for the diagnosis of coronary artery disease by scintigraphy is 99mTc-labeled methoxy-isobutyl-isonitrile (MIBI).^15^ This agent can attach to an unidentified cytosolic protein, or become sequestered in mitochondria.^16^ In vivo, 99mTc-MIBI is picked up by tissues containing a lot of mitochondria, such as cardiac muscle and tumors.^17^ In vitro, 99mTc-MIBI accumulates in cultured myocytes, endothelial cells, and in v-src-transformed, but not in untransformed, NIH 3T3 fibroblasts.^18^

Abnormal activity of 99mTc-MIBI by neoplastic tissues in the lungs has been mentioned previously, but pulmonary uptake in non-neoplastic conditions seems to be extremely infrequent.^19^

Although reports from preclinical and pilot studies are promising, this agent is not presently applied for routine clinical use. We aimed to ascertain the efficacy of 99mTc-MIBI lung scans in the early detection and evaluation of the severity of pulmonary involvement in patients with ILD.

## MATERIALS AND METHODS

### Participants

This study was conducted on 19 patients with a history of ILD who were referred from the lung department of a university hospital to our research center. Participants excluded from this study included those with a history of chronic respiratory disease, chronic cardiac failure, lung cancer, severe pulmonary arterial hypertension, and a recent history or signs of respiratory infection at the time of the study. Also excluded were those with an Ig-A deficiency, heavy smoking, and a history of immune-mediated reactions to blood transfusions. The patients underwent PFTs and HRCT scans followed by 99mTc-MIBI scanning. They were then followed for 1 year. Corticosteroids were not used before scanning. Five patients without lung disease, who were assessed with 99mTc-MIBI scintigraphy for the diagnostic workup of myocardial perfusion, were considered as the control group for 99mTc-MIBI uptake in the lungs.

This study complies with the Declaration of Helsinki and was approved by the Institutional Ethics Committee of Bushehr University of Medical Science. All patients provided their written informed consent prior to the study.

### Severity Grading of Respiratory Complications

Pulmonary function parameters were acquired with a spirometer (ZAN 100, Me, greräte GmbH, Germany). The severity grading of respiratory damage was categorized based on the patient's forced vital capacity (FVC) and forced expiratory volume in the first second (FEV1). Normal respiratory function (grade 1) was described as an FVC ≥ 80% and an FEV1 ≥ 80% of the predicted value. Mild respiratory damage (grade 2) was described as an FVC = 60% to 79% or an FEV1 = 60% to 79% of predicted values; moderate (grade 3) was an FVC = 50% to 59% or an FEV1 = 40% to 59% of predicted values; and severe (grade 4) was an FVC < 50% or an FEV1 < 40% of predicted values.^20^

### High-Resolution Computed Tomography Scan

High-resolution computed tomography images were performed on an electron beam CT machine. All 1.5 mm sections were done at full inspiration both in the supine and prone positions. In all participants, additional expiratory films and prone sections were acquired. Images were photographed at window settings proper for inspecting the lung parenchyma. The scans, together with plain chest radiographs, were assessed by a pulmonary radiologist. The possible patchy ground glass pattern, reticular pattern, emphysema, honeycombing, airway distortion, and bronchial wall thickening appearances were recorded. The parenchymal patterns identified on HRCT were coded, and a score was delineated according to Goldin et al.^21^ For the appraisal of severity, a point value was allocated to each pattern as follows: pure ground-glass opacity, fibrosis (including thickened reticular markings, bronchiectasis, and bronchiolectasis), and honeycombing.^21^

An “extent of disease” score was attained by the percentage of disease extent for each appearance: normal was scored as a 0 with all others scored as follows: 1 < 25%; 2 = 25% to 50%; 3 = 50% to 75%; and 4 > 75%. Three zones were contemplated as follows: zone 1, apex to aortic arch; zone 2, aortic arch to inferior pulmonary veins; and zone 3, inferior pulmonary veins to the diaphragm. The right and left lungs were scored separately.^21^ Lastly, the scores for the severity and extent of the disease were added to acquire a total HRCT score. For example, a participant with a ground-glass appearance in >75% of 6 zones in both lungs was scored with this equation: 6 × 4 = 24 score. Honeycombing in <25% of 3 zones in the right lung and also <25% of zone 1 in the left lung, in 50% to 75% of zone 2 of the left lung, and in 24% to 50% of zone 3 of the left lung but lack of reticular fibrosis in any part of the lung would have a total HRCT score of 33.^21^

### Imaging Protocols

#### 99mTc-MIBI Scintigraphy

Scans were performed with commercially available MIBI kits that were prepared using freshly eluted 99mTc and the labeling efficiency of MIBI was always >95%.

The 99mTc-MIBI imaging was performed using a large field of view gamma camera fitted with a low-energy, all-purpose collimator. Anterior and posterior images of the chest were obtained 20 min and 60 min following the intravenous injection of 370 MBq (10 mCi) 99mTc-MIBI. Images were obtained using a double detector system (ADAC Genesys Malpitas, CA) with a low-energy, all purpose (LEAP) collimator. For 99mTc-MIBI single-photon emission computed tomography (SPECT), a symmetric 15% window was centered at 140 KeV. Images were recorded in a 256 × 256 word matrix on a nuclear medicine computer. To reduce the superimposed scapular and pectoral muscular activities from the field of the lungs, acquisition was performed in the hands-overhead position. In instances of no abnormality on the early images, either additional oblique-lateral views or SPECT images were taken following late planar imaging to enhance lesion detectability. The 99mTc-MIBI scintigraphy assessment of MIBI uptake was done qualitatively (subjectively) and quantitatively. A nuclear medicine specialist evaluated the projections to optimize the quality of the images.

#### Image Analysis

Images were assessed by 2 nuclear medicine physicians who were blinded to all other clinical and imaging information. The interobserver variability was resolved by a consensus. Patients showing an increased uptake in early or delayed images, or both images, were regarded as positive. The radionuclide uptake was characterized as follows: no uptake (−), mild uptake (±), moderate uptake (+), and severe uptake (++).

For semiquantitative interpretation, a region-of-interest (ROI) analysis was carried out on the anterior and posterior views of the lungs and also a region on the deltoid muscle. The mean count per pixel of the same size of upper, middle, and lower regions of each lung, and the heart were subtracted from the deltoid region yielding net counts per pixel. The geometric mean of these ROIs was determined. The ratio of the mean counts of these ROIs and the counts of the ROI over the heart divided by 2 gave a value for each patient that represented the disease severity using 99mTc-MIBI scintigraphy (MIBI score).

### Statistical Analysis

A 2-tailed *t* test was used to compare the mean values between groups. The continuous variables are expressed as the mean ± standard deviation (SD), and categorical variables as the absolute values and percentages. The distribution of the variables was assessed using probability plots and the Shapiro-Wilk test and they were not fit to a Gaussian distribution. The Mann–Whitney *U* test was applied for quantitative comparisons of MIBI scores between the patient and control groups. The Spearman rank test was used for correlations. A *P* value of < 0.05 was considered statistically significant. Statistical analysis was performed using an IBM computer and PASW software, version 18.0 (SPSS, Inc, Chicago).

## RESULTS

This study included 10 males and 9 females (mean age: 49.33 ± 5.42 years; range: 42–56 years) who had a history of ILD (Table 1). The study population also included 5 patients who referred for an evaluation in a cardiac study. None of the subjects had a history of suspected or documented lung abnormalities.

**TABLE 1:**
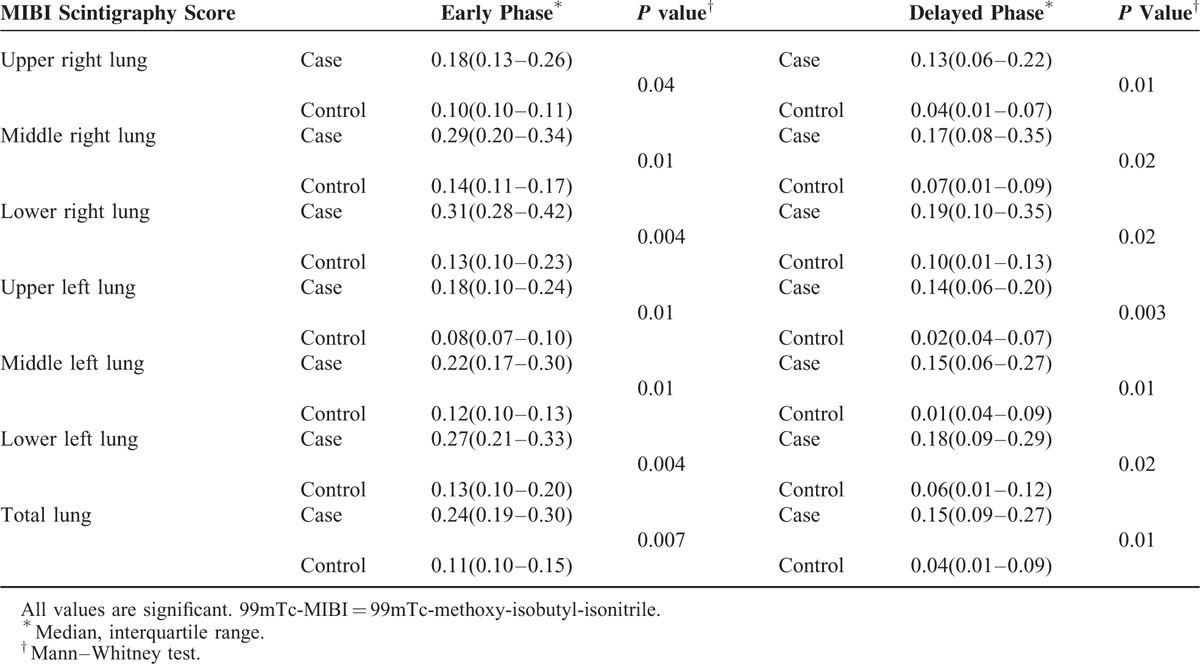
The Comparison of 99mTc-MIBI Scintigraphy Scores Between Patients and Control Groups in Different Lung Regions

**FIGURE 1:**
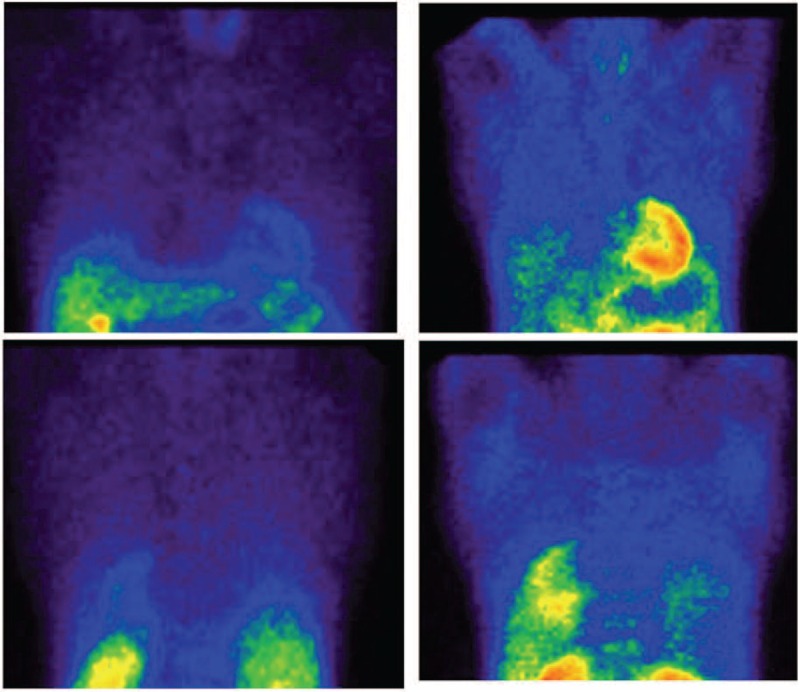
(A) There was significant activity in the lung fields in the early views (left column) of 99mTc-MIBI scintigraphy of a 56-year-old man, which persisted over the course delayed views up to 4 h (right column). The early MIBI score was 0.25 and the delayed MIBI score was 0.12. (B) HRCT scan of the same patient (score 14). HRCT = high-resolution computed tomography, 99mTc-MIBI = 99mTc-methoxy-isobutyl-isonitrile.

**FIGURE 2:**
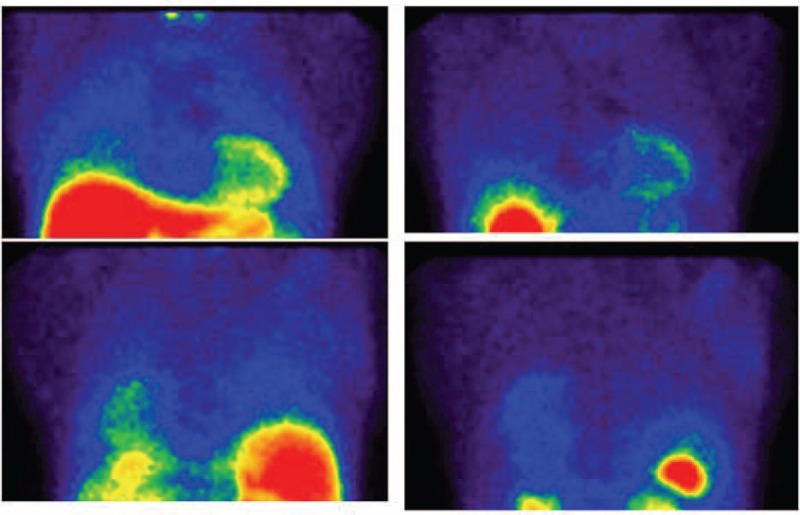
(A) There was significant activity in the lung fields in the early views (left column) of 99mTc-MIBI scintigraphy of a 54-year-old man, which persisted over the course delayed views up to 4 h (right column). The early MIBI score was 0.39 and the delayed MIBI score was 0.35. (B) HRCT scan of the same patient (score 23). HRCT = high-resolution computed tomography, 99mTc-MIBI = 99mTc-methoxy-isobutyl-isonitrile.

**FIGURE 3:**
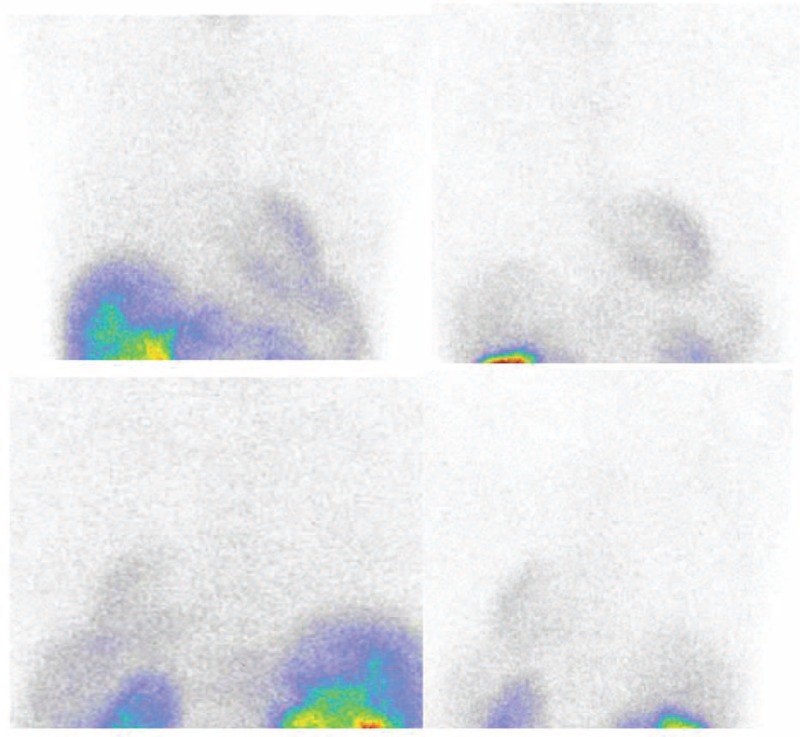
There is no remarkable uptake on the early views (left column) and delayed views of 99mTc-MIBI scintigraphy (right column). The early MIBI score was 0.11 and the delayed MIBI score was 0.01.99mTc-MIBI = 99mTc-methoxy-isobutyl-isonitrile.

Nine patients showed severe activity on the 99mTc-MIBI scan, 4 patients had moderate uptake, and 6 patients had mild activity (Figures 1 and 2). In the qualitative analysis, the 99mTc-MIBI scans in the 5 control patients showed no significant uptake in the lungs (Figure 3).

All 19 ILD patients demonstrated a strong increase in 99mTc-MIBI uptake in the lungs compared to the control group (Table 1). Scores for the 99mTc-MIBI scans were higher in the patient group in both the early phase (0.24[0.19–0.31] vs 0.11[0.10–0.15], *P* < 0.05) and the delayed phase (0.15[0.09–0.27] vs 0.04[0.01–0.09], *P* < 0.05) compared with the control group. A positive correlation was detected between the 99mTc-MIBI scans and the HRCT scores (Spearman's correlation coefficient = 0.65, *P* < 0.02) in the early phase but not in the delayed phase in the patients (*P* > 0.14).

PFT grading of 7 patients were in grade 1 (normal); 3 were in grade 2 (mild); 3 were in grade 3 (moderate); and 6 were in grade 4 (severe). 99mTc-MIBI scan scores were not significantly correlated with PFT findings and also age (*P* value > 0.05).

The association among 99mTc-MIBI scans with HRCT patterns including ground glass opacity, reticular fibrosis, and honeycombing was not significant (*P* > 0.05). Additionally, we did not observe a significant association between 99mTc-MIBI scan scores and HRCT scores in 3 classified zones (*P* > 0.05).

Five patients died and 14 patients were still alive over the 1-year follow-up period. Also, there was a significant difference between the uptake intensity of 99mTc-MIBI and the outcome in the early phase (dead: 0.32[0.29–0.43] vs alive: 0.21[0.18–0.24], *P* < 0.05) and the delayed phase (dead: 0.27[0.22–0.28] vs alive: 0.10[0.07–0.19], *P* < 0.05) (Table 2).

**TABLE 2:**
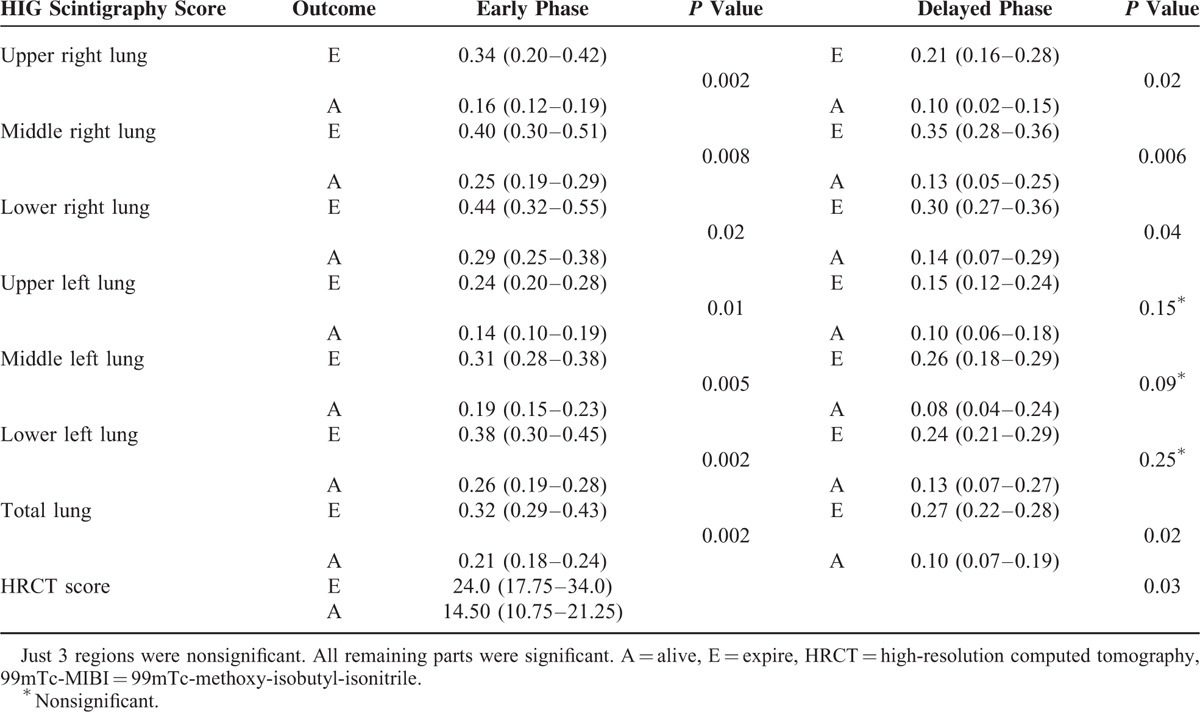
The Comparison of 99mTc-MIBI Scintigraphy and HRCT Scores Between Living Patients and Those Who Died During the Follow-Up Period

The washout rate (WR) was ∼40 min starting from 20 min up to 60 min and demonstrated a significant difference in our 2 study groups (ILD: 46.61[15.61–50.39] vs NL: 70.91[27.09–116.36], *P* = 0.04). The WR was acquired as follows: (early MIBI score – delayed MIBI score)/early MIBI score × 100.

## DISCUSSION

The most important finding of this study underscores the advantages of 99mTc-MIBI scintigraphy as a rapid and precise imaging tool for the assessment of the inflammatory process in the lungs. Although there was a total association between 99mTc-MIBI scintigraphy and HRCT scores, there were few patients with higher 99mTc-MIBI scores in a lower HRCT group compared to a higher HRCT group. This might imply that anatomical imaging modalities are often improper for the early detection of inflammation due to the consideration of only morphological changes.^22,23^ However, a nuclear modality can easily distinguish an inflammatory process because it is based on functional processes that are morphologically indistinguishable.^24^

In nuclear medicine fields, some radiopharmaceuticals have been proposed for the detection of active interstitial pulmonary lesions. For example, 67Ga-citrate is used in the assessment of sarcoidosis and lung infections. A high uptake of 67Ga-citrate in the lung is reported in patients with severe pulmonary fibrosis and dyspnea; however, it is not correlated with the laboratory indices of the disease.^12^ Scintigraphy with 99mTc-DTPA aerosol is also a helpful modality in the study of pulmonary epithelial permeability in patients with connective tissue disease (CTD). In a recent study in patients with mixed CTD, there was a diminished clearance time of 99mTc–DTPA, which was improved after therapy.^25^ On the other hand, Antoniou et al^26^ reported that, in patients with idiopathic pulmonary fibrosis, 99mTc-DTPA scintigraphy findings are not related to HRCT and PFTs. Furthermore, an increased uptake was noted in 111In–octreotide lung scans of patients with idiopathic pulmonary fibrosis. Thus, the usefulness of radiopharmaceuticals in the detection and evaluation of the severity of pulmonary involvement in CTD is not yet established.^4^

The use of 99mTc-MIBI scanning is now widely used for myocardial imaging. Additionally, it appears to have a role in the diagnosis and staging of tumors, and in the noninvasive assessment of ischemia in patients with peripheral vascular diseases.^27^ Results from the present study indicate that the lung uptake of 99mTc-MIBI was well-correlated with markedly abnormal in patients with ILD who had clinical and/or radiologic evidence of pulmonary involvement.

Several 99mTc-MIBI uptake mechanisms are thought to contribute to its accumulation in the lung.^14,28^ On one hand, it is thought to be due to an increased vascularity and vascular permeability at the site of inflammation, and being lung fibroblasts, pulmonary vascular endothelial cells, or inflammatory cells infiltrating the parenchymal interstitium.^29^ On the other hand, it could go back to the chemical characteristics of radiopharmaceuticals, the cationic charge, the lipophilic properties, and the negative transmembrane potentials generated in the cytoplasm and mitochondria of metabolically active cells.^30^

The lung uptake in patients who underwent a myocardial perfusion imaging with suspected or confirmed cardiac disease correlate well with the degree and severity of left ventricular systolic dysfunction and angiographic coronary artery disease.^16^ In the absence of cardiac disease, as with our patient group, these appearances may imply the presence of underlying pulmonary processes. Furthermore, some of these causes may include chronic smoking,^31^ chemical pneumonitis,^32^ atypical pneumonias (*P. carinii*, MAC), lymphocytic interstitial pneumonia (LIP), and interstitial pneumonitis secondary to CTD,^29^ all of which were ruled out in our study. In the absence of these situations, the radiotracer uptake in the lungs may most likely represent ILD. However, sole radiotracer uptake in the diagnosis of ILD is nonspecific; thus, a tissue biopsy and histologic examination are reasonable to verify the ILD diagnosis.

In the current investigation, there was no association between 99mTc-MIBI scores and PFT results, which may indicate the significance of 99mTc-MIBI scintigraphy in the assessment of the severity of pulmonary involvement.

Alternatively, it is reported that 111In-octreotide uptake in the lungs is correlated with the fibrosis score in HRCT, but not with the ground-glass score in the idiopathic pulmonary fibrosis patients.^4^ In contrast, there was no association between the patterns HRCT and MIBI scores in the present study, which may be due to the different population and low numbers of included cases in our study.

We also demonstrated the value of 99mTc-MIBI pulmonary scintigraphy in its ability to differentiate active from inactive pulmonary tuberculosis in 19 patients with history of pulmonary tuberculosis.^33^

Likewise, Richard et al performed a pilot study to assess the clinical significance of abnormal lung uptake of 99mTc-MIBI in 16 patients with systemic sclerosis. The intensity of uptake in the lungs was correlated with the extent of maximal skin induration and with radiologic evidence of ILD, but not with other clinical or laboratory factors of disease activity or the extent of pulmonary involvement.^19^

What's more, the 99mTc-MIBI scores in the present study were significantly different in patients who were still living compared to those who had died during the follow-up period, which may represent promising results for the differentiation of active and inactive lesions in future human studies. In terms of a prognosis role for 99mTc-MIBI scans, few studies in patients with lung cancer have been reported, and these have concluded that 99mTc-MIBI scans can predict the survival of lung cancer patients.^34^ However, due to small endpoints in our prospective noncancer investigation, further larger and well-designed studies are necessary.

We depicted a lesser washout rate in ILD patients compared with the healthy group (*P* < 0.05). This means that 99mTc-sestamibi elimination rates in ILD patients from 20 min to 60 min were less than the normal rates. In line with this, Ruparelia et al reported pulmonary elimination rates of inhaled 99mTc-sestamibi radioaerosol in 26 in healthy smokers and 15 nonsmokers plus 7 patients with COPD (all ex-smokers) and 3 with ILD (all nonsmokers).^35^ The 99mTc-sestamibi elimination rates in the ILD patients ranged from 6 to 30 min, were not accelerated, and were 0.23, 0.32, and 0.35% min^−1^.^35^ However, the type of usage and washout period times were different compared with our investigation times.

We also worked on 99mTc-IgG lung scintigraphy in the assessment of pulmonary involvement in ILD in 8 patients with ILD and 6 control participants.^36^ All 8 ILD patients showed a strong increase in 99mTc-IgG uptake in the lungs compared to the control patients. In the ILD patients, a statistically significant positive correlation was detected between 99mTc–IgG scans and HRCT scores (Spearman's correlation coefficient = 0.92, *P* < 0.008). Most findings in a recent study with 99mTc-MIBI are similar to the 99mTc-IgG study.^36^

In the comparison of both radiopharmaceuticals (99mTc-MIBI vs 99mTc-IgG) in the detection of pulmonary involvement in ILD groups, we should point out that 99mTc-MIBI is widely available and less expensive with a better quality compared with 99mTc-IgG.

In the present study, we found differences between the MIBI indices of the patient and control groups in the early and delayed 99mTc-MIBI scans. Therefore, it seems that the 20-min scan is sufficient and the delayed scintigraphy is not necessary.

In terms of radiation burden, typical effective radiation dose from chest CT scan is ∼8.00 mSv and from 370 MBq (10 mCi) 99mTc-MIBI is ∼3.3 mSv.^37,38^ It is essential to note that these are only typical values. Radiation doses vary for each person due to differences in x-ray machines and their settings, the amount of radionuclide given in nuclear medicine techniques, and the patient's metabolism.^38^

In summary, both 99mTc-MIBI and HRCT scans are worthwhile in the assessment of the activity of pulmonary involvement in ILD, particularly when the therapeutic response is not satisfactory. These scans help in the decision for early treatment to lessen patient morbidity and mortality as well as in the prevention of disease aggravation.

Although our study revealed good insight into using 99mTc-MIBI as compared with clinical and radiological assessments, it should be mentioned that it has some limitations. The main limitations of this study were the small sample size and the absence of lung biopsies in the participants as a gold standard test, both of which may have influenced the results of this study. Nevertheless, we did consider a mixed clinical presentation, radiological modalities, and follow-up evaluation to alleviate this deficiency. Our results should be corroborated in a larger and well-designed study.

## CONCLUSIONS

The present study demonstrated that a 99mTc-MIBI lung scan might distinguish the severity of pulmonary involvement in early views, which correlated well with the HRCT findings. These results also revealed that 99mTc-MIBI lung scans might be used as a complement to other diagnostic and clinical examinations in terms of functional information in ILD. However, further investigations are strongly required.
